# Time and patient journey to axial spondyloarthritis diagnosis: a retrospective study in French primary care

**DOI:** 10.1093/rheumatology/keaf642

**Published:** 2026-03-15

**Authors:** Clément Prati, Arnaud Constantin, Emmanuelle Dernis, Jacques Eric Gottenberg, Marc Rozenblat, Elise Arnee, Marie Ducros, Cheikh Tamberou, Anneleen Vyncke, Julie Gandrup Horan

**Affiliations:** Department of Rheumatology, Besancon University Hospital, Besancon, France; Rheumatology, Purpan Hospital and Paul Sabatier Toulouse III University, Toulouse, France; Department of Rheumatology, Caen University Hospital, Caen, France; Rheumatology Department, Hôpitaux Universitaires de Strasbourg, Université de Strasbourg, Strasbourg, France; Centre Coralis, Ozoir La Ferriere, France; GERS DATA, Patient Data France, Boulogne-Billancourt, France; GERS DATA, Patient Data France, Boulogne-Billancourt, France; GERS DATA, Patient Data France, Boulogne-Billancourt, France; UCB, Colombes, France; UCB, Slough, UK

**Keywords:** delivery of health care, observational studies, primary care rheumatology, routinely collected data, spondyloarthritis

## Abstract

**Objectives:**

Axial spondyloarthritis (axSpA) diagnoses are often delayed, which is associated with poorer patient outcomes. To improve understanding of diagnostic delay in France, we describe the time and patient journey to axSpA diagnosis in primary care.

**Methods:**

This retrospective consecutive case series of adult axSpA patients in France (January 2000–August 2023) analysed primary care data from The Health Improvement Network^®^ (THIN) database. Key study outcomes were time from earliest GP-recorded back pain diagnosis to axSpA diagnosis, and number and type of back pain episodes in this period. Other variables included axSpA symptoms and comorbidities at back pain and axSpA diagnosis, and healthcare resource utilization (HCRU).

**Results:**

Of 7313 adult patients diagnosed with axSpA eligible for inclusion, 4402 had a prior back pain diagnosis. Mean time from earliest back pain to axSpA diagnosis was 6.3 years. Mean number of back pain episodes recorded prior to axSpA diagnosis was 6.1; 17.3% of patients experienced ≥10 episodes. Lower back pain was the most common (47.4% of diagnoses). The proportion of patients experiencing >1 axSpA symptom or comorbidity increased from 1.9% or 23.7% at earliest back pain diagnosis to 8.9% or 50.7% at axSpA diagnosis, respectively. HCRU was high; mean number of primary care consultations per patient per year between earliest back pain and axSpA diagnosis was 7.0.

**Conclusions:**

Patients in France wait over 6 years for an axSpA diagnosis. This research may raise awareness of the patient journey to axSpA diagnosis and support development of a primary care flagging strategy to identify patients with suspected axSpA earlier.

Rheumatology key messages

- In France, patients wait 6.3 years, on average, from earliest back pain to axSpA diagnosis.
- Symptoms and comorbidities increase from time of earliest back pain diagnosis to axSpA diagnosis.
- Earlier axSpA diagnosis may potentially lower burden of axSpA on patients, healthcare systems and society.

## Introduction

Axial spondyloarthritis (axSpA) is a chronic, inflammatory disease affecting the sacroiliac joints and spine, with a prevalence of ∼0.3–0.7% worldwide [1, 2] and 0.3–0.4% in France [3–5]. The axSpA disease spectrum encompasses non-radiographic and radiographic axSpA, depending on the presence of definitive structural damage of the sacroiliac joints on radiographs [2, 6, 7]. It is characterized by chronic lower back pain, stiffness, fatigue and sometimes peripheral musculoskeletal and extra-musculoskeletal manifestations. Due to these symptoms, patients with axSpA suffer from impaired quality of life [2, 8, 9].

Patients with axSpA typically experience first onset of disease aged ∼20–34 years, with back pain being a common initial symptom [10, 11]. Most patients first present to primary care physicians or non-specialist healthcare professionals (HCPs), and receive multiple consultations prior to diagnosis [9, 12]. Diagnosis is based on a combination of symptoms, physical examination, family history and tests (i.e. laboratory, imaging) [2].

Early diagnosis and intervention are crucial for optimal axSpA management to avoid disease progression [9, 13]. Despite this, delayed diagnosis remains a key challenge in axSpA care, with a mean global and European estimated delay of 6.7 and 7.4 years, respectively, and little improvement observed over recent years [12–14]. Although diagnostic delay is well documented globally, variation in how this is estimated and reliance on patients self-reporting the time of symptom onset has led to significant disparity in the extent of the delay reported within and between countries [12, 14–16].

Causes of diagnostic delay include difficulty in distinguishing inflammatory back pain caused by axSpA from other types of back pain, and a lack of awareness amongst HCPs of the key features and risk factors of axSpA [13, 17, 18]. Patient reluctance to approach HCPs at first symptom occurrence and long wait times for specialist consultations may also contribute [18]. Other factors previously associated with longer diagnostic delay include female sex and HLA-B27 negativity [13].

The consequences of diagnostic delay for patients with axSpA are significant and include higher disease activity resulting in spinal damage and fusion, and poorer quality of life and treatment response [9, 13]. Diagnostic delay also leads to economic burden, and is associated with greater work impairment and increased healthcare resource utilization (HCRU) [9]. There is therefore a clear unmet need to diagnose patients with axSpA earlier in the course of their disease, ideally when they first present to primary care with symptoms.

To raise awareness and improve understanding of the current extent of diagnostic delay in France, and to inform future strategies to reduce this, we aimed to describe the time and patient journey to axSpA diagnosis in primary care in France using data from The Health Improvement Network (THIN)^®^ database [19].

A plain language summary of this article is available as a Supplementary Material.

## Methods

### Study design and participants

**Figure 1.**
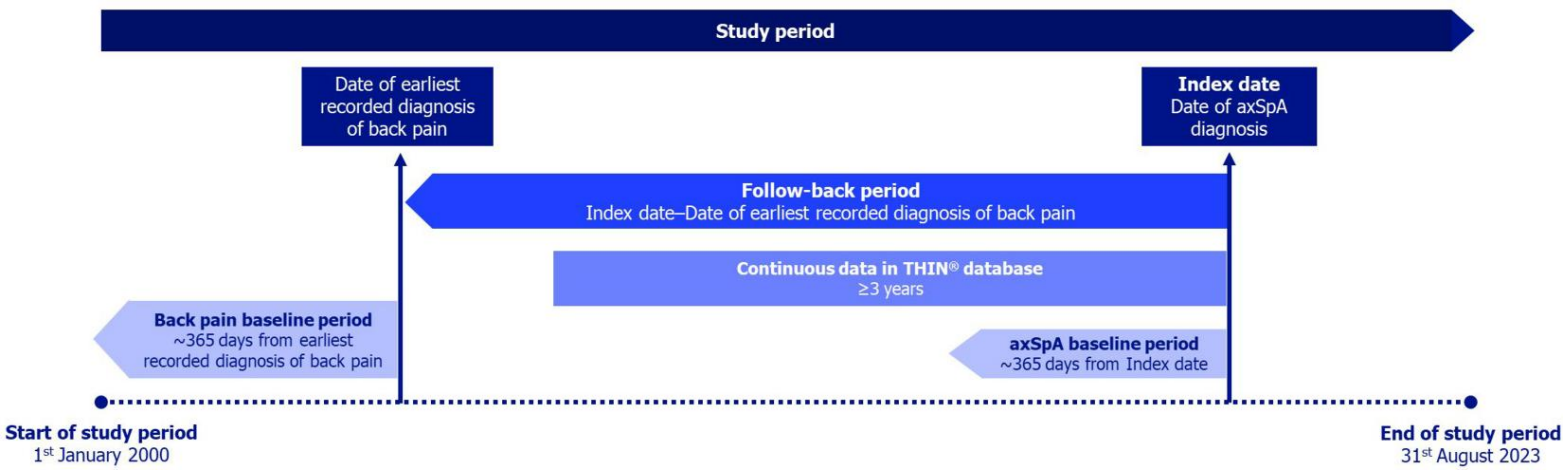
Study design. axSpA: axial spondyloarthritis; THIN: The Health Improvement Network

This study was a retrospective consecutive case series of adults with an axSpA diagnosis in France, based on analyses of primary care data from the French THIN database from 1 January 2000 to 31 August 2023 (Fig. 1). The date of the first diagnostic code for axSpA recorded in the database by a general practitioner (GP), representing the time of axSpA diagnosis, was defined as the ‘index date’.

Participants were included if they had an axSpA diagnosis between 1 January 2010 and 31 August 2023, and were ≥18 years at the index date with no missing age or sex data (Supplementary Fig. S1). To avoid potential underestimation of time to diagnosis caused by including newly-registered participants reporting pre-existing axSpA diagnoses, ≥3 years of continuous data in the THIN database prior to the index date was required. Participants were excluded if they had a new RA or PsA diagnosis in the year prior to the index date.

For patients with a recorded back pain diagnosis prior to axSpA diagnosis, the follow-back period extended from the index date to the earliest recorded back pain diagnosis. Both the index date and date of earliest back pain diagnosis had baseline periods consisting of the prior 365 days (Fig. 1).

To describe the time and patient journey to axSpA diagnosis, multiple subgroups were defined (Supplementary Fig. S1). The ‘no back pain’ and ‘back pain’ subgroups included participants *without* and *with* a recorded back pain diagnosis prior to axSpA diagnosis, respectively. The ‘axSpA indicators’ subgroup included participants with a recorded diagnosis of back pain *and/or* any other axSpA-related feature prior to axSpA diagnosis. AxSpA-related features were those described in the Assessment of SpondyloArthritis international Society (ASAS) classification criteria (e.g. peripheral arthritis, enthesitis, dactylitis, uveitis, psoriasis and inflammatory bowel disease) [20] and available in the THIN database. The back pain subgroup was the primary focus for analyses.

To assess HCRU, the back pain subgroup was further split based on completeness of medical reimbursement history (see ‘Data sources’ for more information): participants with a ‘complete’ reimbursement history between 2014 and 2023 or ≥5 years of continuous reimbursement data prior to axSpA diagnosis, and participants with ‘any’ reimbursement history (i.e. with gaps of any length, including a ‘complete’ reimbursement history) between 2014 and 2023.

### Data sources

The French THIN database contains anonymized patient data from 2000 GPs in retail practice, where ‘retail practice’ refers to the community-based, non-hospital patient care activities of GPs. Collected data include demographic and medical data (e.g. diagnoses, tests, imaging or drug prescriptions, and medical or paramedical reimbursement data), linked to individuals via unique identification numbers.

Diagnoses were determined via International Classification of Diseases 10th Revision (ICD-10) codes, which were mapped to corresponding Base Claude Bernard (BCB) codes recorded within the THIN database (Supplementary Tables S1 and S2). A broad and inclusive range of back pain codes relevant to axSpA, including multiple pain aetiologies and locations, were considered and validated by a panel of clinical experts (a subset of the authors).

In French primary care, a 12-month history of HCRU is extracted from the national health insurance database and linked to the THIN database on an individual basis, upon request by a GP. These data were only available from 2014 onwards, and to have continuous reimbursement history, the GP must have requested HCRU data ≥1 time per year. HCRU-related costs were based on national pricing reference tables.

### Variables

#### Study participants

Baseline period patient characteristics included age, sex, length of continuous medical history and Charlson Comorbidity Index (CCI) [21].

#### Key variables

Key study variables were time to diagnosis from earliest documented back pain to axSpA diagnosis, and the number and type of back pain episodes (i.e. occurrences, on separate days/visits) recorded prior to axSpA diagnosis.

Additionally, the burden of musculoskeletal and extra-musculoskeletal symptoms of axSpA, and comorbidities, were assessed at time of earliest back pain diagnosis and at axSpA diagnosis. Musculoskeletal symptoms included enthesitis and dactylitis; extra-musculoskeletal symptoms included uveitis, psoriasis, Crohn’s disease and ulcerative colitis. Comorbidities were selected by a panel of clinical experts based on relevance to patients with axSpA.

HCRU was reported for each year between earliest back pain and axSpA diagnosis as the number and type of consultations (for any reason), laboratory tests (CRP or ESR; HLA-B27 testing was of interest but not available within the dataset), imaging tests (CT, MRI, US or X-ray of spine, pelvis and hips) and prescriptions (chosen based on relevance to early-stage axSpA), and the number and cumulative duration of sick leave episodes. HCRU related to medical transport and hospitalization were not considered due to a lack of available data in the THIN database. HCRU and associated costs were reported per patient per year (PPPY), corresponding to:


$$\frac{\mathrm{Total}\;\mathrm{cost}\;\mathrm{of}\;\mathrm{healthcare}\;\mathrm{resource}\;\mathrm{use}\;}{\mathrm{Duration}\;\mathrm{of}\;\mathrm{follow}\;\mathrm{up}\;\mathrm{in}\;\mathrm{months}\;}\;\times \;12$$

#### Other variables

Other study variables were time to diagnosis from earliest documented back pain or axSpA-related feature to axSpA diagnosis, and the number and type of back pain episodes and axSpA-related features recorded prior to axSpA diagnosis.

### Statistical analysis

Descriptive statistical analyses included mean and S.D. for continuous variables, and number and proportion of patients for categorical variables. No data were missing for the variables reported.

The difference in time to diagnosis between males and females was assessed using an independent Student’s *t*-test. The difference in prevalence of symptoms of axSpA or comorbidities at earliest back pain diagnosis and axSpA diagnosis was assessed using McNemar’s test.

### Sensitivity analysis

To assess the impact of focusing on earliest individual back pain diagnoses, rather than chronic back pain, time to diagnosis was also measured for patients with ≥2 back pain diagnoses recorded ≥90 days apart. Time to diagnosis was measured from the earliest of the two back pain diagnoses to axSpA diagnosis.

Additionally, to assess the impact of the inclusion criteria of ≥3 years of continuous data prior to axSpA diagnosis, time to diagnosis was reported for two subgroups with: (1) no minimum requirement for continuous data in the THIN database and (2) ≥7 years of continuous data coverage.

### Ethics

As the study analysed a retrospective anonymized dataset, no ethical review and no informed consent from patients were needed. However, the study protocol was reviewed by a scientific steering committee and the data owner. The work on the dataset conformed to all General Data Protection Regulation (GDPR) requirements.

## Results

### Study participants

Of the 22 800 patients with a new axSpA diagnosis between 1 January 2010 and 31 August 2023 recorded in the THIN database, 7313 met the criteria for inclusion (Supplementary Fig. S1). Among these:

- 4826 (66.0%) had a back pain diagnosis and/or an axSpA-related feature diagnosis recorded prior to axSpA diagnosis (axSpA indicators subgroup).
- 4402 (60.2%) had a prior back pain diagnosis (back pain subgroup).
- 1430 (19.6%) had both a prior back pain diagnosis and an axSpA-related feature diagnosis.
- 2911 (39.8%) did not have a back pain diagnosis prior to axSpA diagnosis (no back pain subgroup; Supplementary Table S3).

In the back pain subgroup, mean (S.D.) age was 40.3 (13.9) and 46.7 (14.2) at earliest back pain diagnosis and axSpA diagnosis, respectively. At axSpA diagnosis, 56.5% of participants were female, mean length of continuous medical history was 10.4 (4.9) years and 21.2% of participants had a CCI score ≥3 (Table 1).

**Table 1:** Demographics and characteristics among patients with back pain recorded prior to axSpA diagnosis.

	Patients with back pain prior to axSpA diagnosis (*N* = 4402)	
	At back pain diagnosis	At axSpA diagnosis
Age, years, mean (S.D.)	40.3 (13.9)	46.7 (14.2)
Sex, *n* (%)		
Male	1913 (43.5)	1913 (43.5)
Female	2489 (56.5)	2489 (56.5)
Length of continuous medical history,^a^ years, mean (S.D.)	–	10.4 (4.9)
CCI,^b^ *n* (%)		
0	2691 (61.1)	1634 (37.1)
1–2	1324 (30.1)	1833 (41.6)
3–4	319 (7.2)	650 (14.8)
>4	68 (1.5)	285 (6.5)

a The length of continuous medical history available in the THIN^®^ database, following back from the date of axSpA diagnosis (i.e. index date).

b CCI data are reported across the baseline periods (365 days prior) for the date of back pain diagnosis and date of axSpA diagnosis, respectively.

axSpA: axial spondyloarthritis; CCI: Charlson Comorbidity Index

### Time to diagnosis from earliest recorded back pain

**Figure 2.**
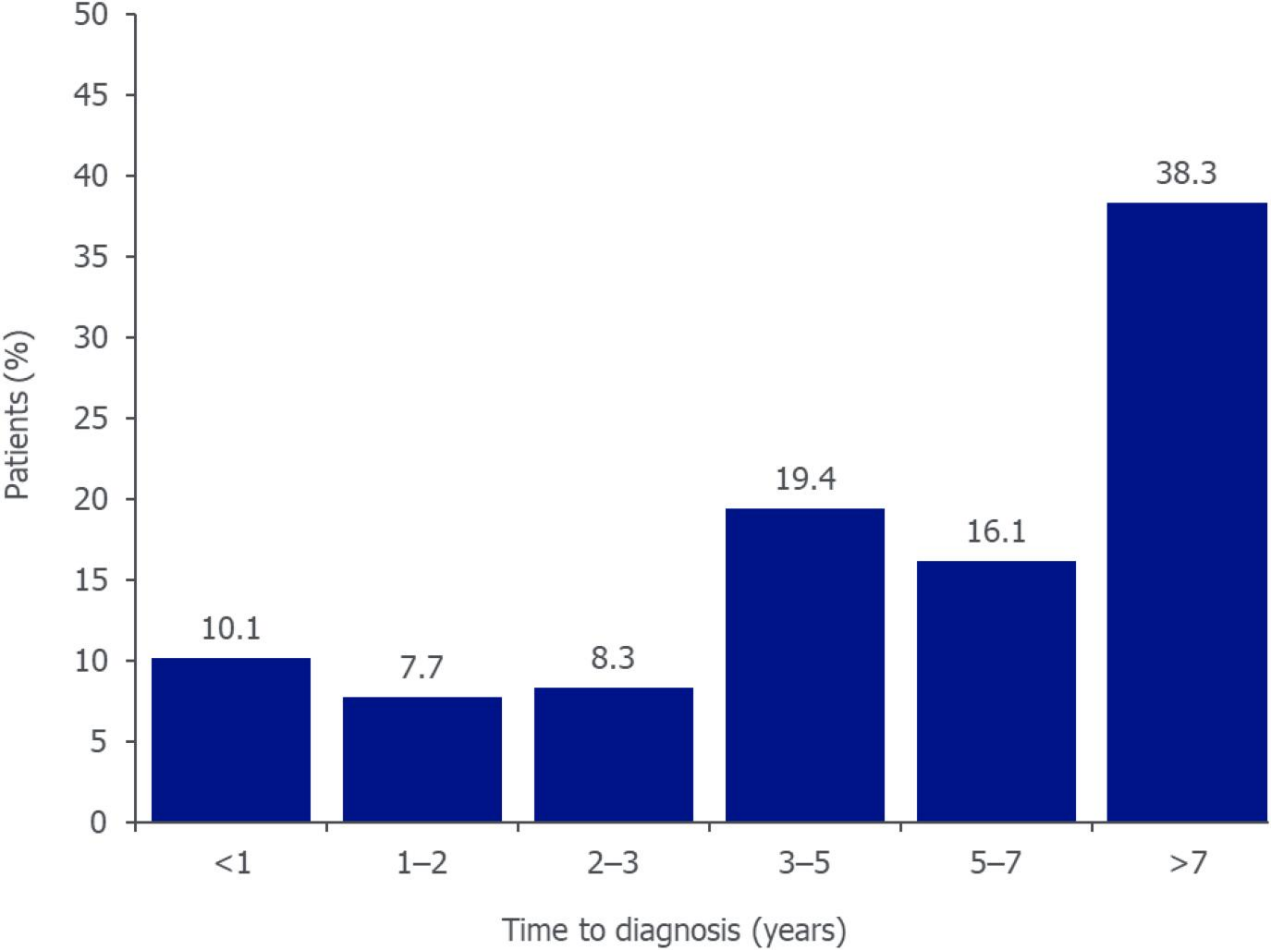
Time to axSpA diagnosis among patients with a previous diagnosis of back pain. Reported for the back pain subgroup (*N* = 4402). Time to diagnosis was calculated based on the length of time to axSpA diagnosis from the earliest documented back pain diagnosis. The >7-year final category was selected given the average diagnostic delay for axSpA of approximately 7 years reported in the literature. Due to rounding, data labels may not total 100%. axSpA: axial spondyloarthritis

Mean (S.D.) time to diagnosis of axSpA for the back pain subgroup (*N* = 4402) was 6.3 (4.5) years (median: 5.5; interquartile range: 2.9–9.3). Time to diagnosis was >7 years in 38.3% of patients (Fig. 2).

Mean time to diagnosis was 6.4 (4.4) and 6.2 (4.5) years for females (*N* = 2489) and males (*N* = 1913), respectively (no significant difference: *P* = 0.12).

Mean (S.D.) number of back pain episodes recorded by GPs on different visits prior to axSpA diagnosis was 6.1 (8.5); 17.3% of patients had ≥10 episodes.

**Figure 3.**
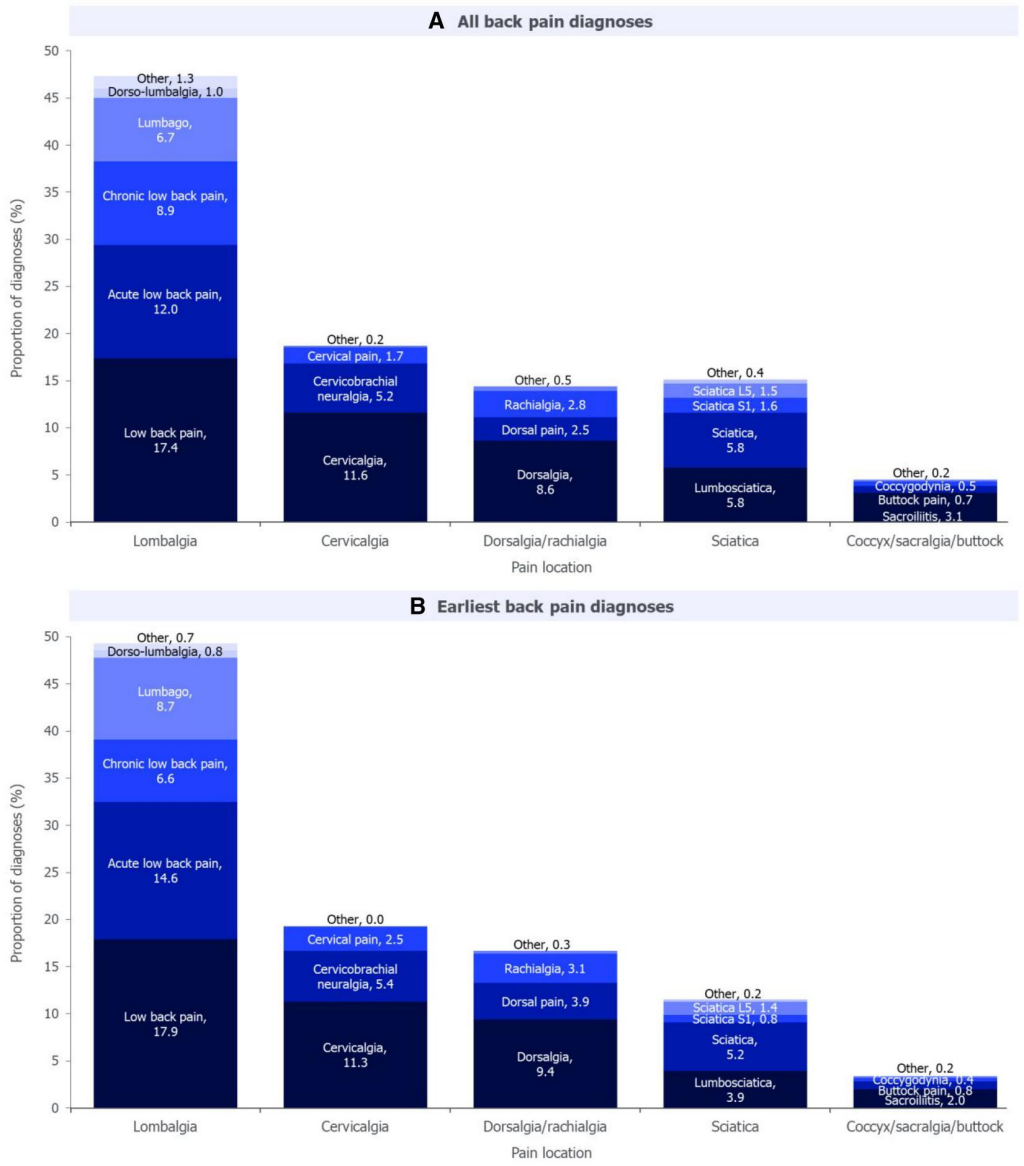
Distribution of location and type of all and earliest back pain diagnoses prior to axSpA diagnosis. Reported for the back pain subgroup (*N* = 4402). Data are reported as (A) a proportion of the total number of back pain diagnoses (*N* = 26 857) and (B) a proportion of the total number of earliest back pain diagnoses (*N* = 4906). A single patient could have two ‘earliest’ diagnoses on the same date. The sum of component values may not precisely match overall values due to rounding. Colours are used to differentiate categories, aiding in visual distinction rather than indicating equivalence. axSpA: axial spondyloarthritis

Of the 26 857 total back pain diagnoses recorded prior to axSpA diagnosis in the back pain subgroup, lower back pain (lombalgia) was the most common site, accounting for 47.4% of diagnoses (Fig. 3A). Neck pain (cervicalgia) and thoracic spinal pain (dorsalgia) were the second and third most common, accounting for 18.7% and 14.4% of diagnoses, respectively. Of the earliest back pain diagnoses, the most common types and locations of back pain were the same as those for all back pain diagnoses (lower back pain: 49.2%; neck pain: 19.2%; thoracic spinal pain: 16.7%; Fig. 3B).

### Proportion and distribution of symptoms of axSpA and comorbidities

**Figure 4.**
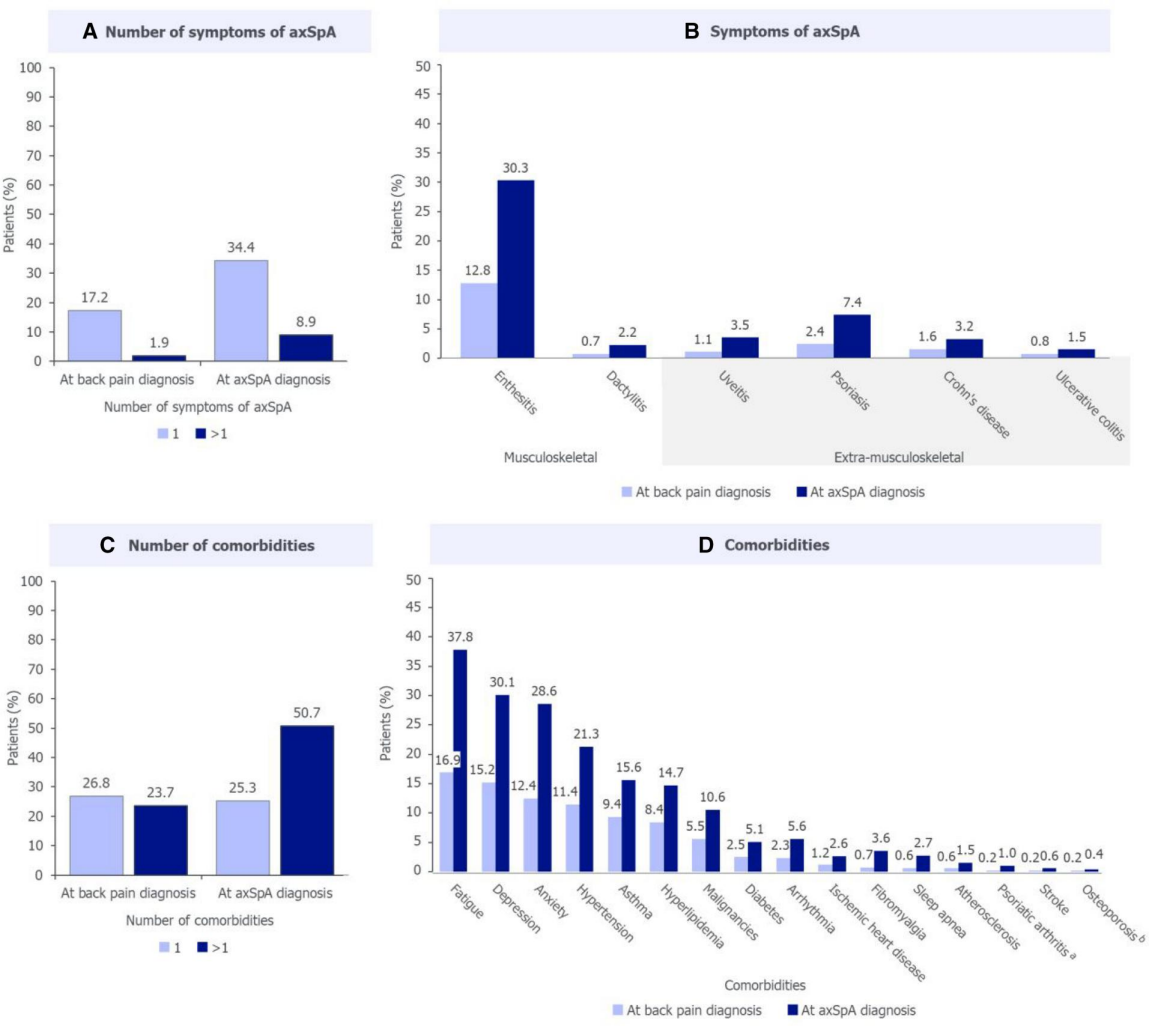
Non-back pain-related symptoms of axSpA and comorbidities at earliest back pain diagnosis and at axSpA diagnosis. Reported for the back pain subgroup (*N* = 4402). Symptoms of axSpA reported here do not include those related to back pain. Significant difference in prevalence at back pain diagnosis and at axSpA diagnosis reported for all comorbidities or symptoms of axSpA assessed (McNemar’s test; *P* ≤ 0.05), except for osteoporosis. ^a^Although patients with a new diagnosis of PsA in the year prior to axSpA diagnosis were excluded from analyses, patients with a previous diagnosis of PsA before this timepoint were included. ^b^No significant difference in osteoporosis prevalence at back pain diagnosis and at axSpA diagnosis. axSpA: axial spondyloarthritis

Among the back pain subgroup, the proportion of patients experiencing >1 musculoskeletal or extra-musculoskeletal symptom of axSpA increased from 1.9% at earliest back pain diagnosis to 8.9% at axSpA diagnosis (Fig. 4A). Of these symptoms, enthesitis (including codes listed in Supplementary Table S4) showed the greatest increase (17.5%), followed by psoriasis (5%; Fig. 4B).

The proportion of patients experiencing >1 comorbidity increased from 23.7% at earliest back pain diagnosis to 50.7% at axSpA diagnosis (Fig. 4C). Fatigue showed the greatest increase (20.9%), followed by anxiety (16.2%; Fig. 4D). The difference in prevalence at earliest back pain diagnosis and at axSpA diagnosis was significant for all symptoms of axSpA and comorbidities assessed (*P* ≤ 0.05), except for osteoporosis.

### HCRU and costs

#### Subgroup selection

Within the back pain subgroup, baseline characteristics, musculoskeletal or extra-musculoskeletal symptoms of axSpA, and comorbidity burden were assessed at earliest back pain and axSpA diagnosis, for patients with a complete reimbursement history (*N* = 402) and patients with any reimbursement history (*N* = 1513).

Based on a greater similarity to the wider back pain subgroup, the subset of patients with any reimbursement history was chosen to describe HCRU and costs (Supplementary Table S5 and Fig. S2).

#### HCRU

**Figure 5.**
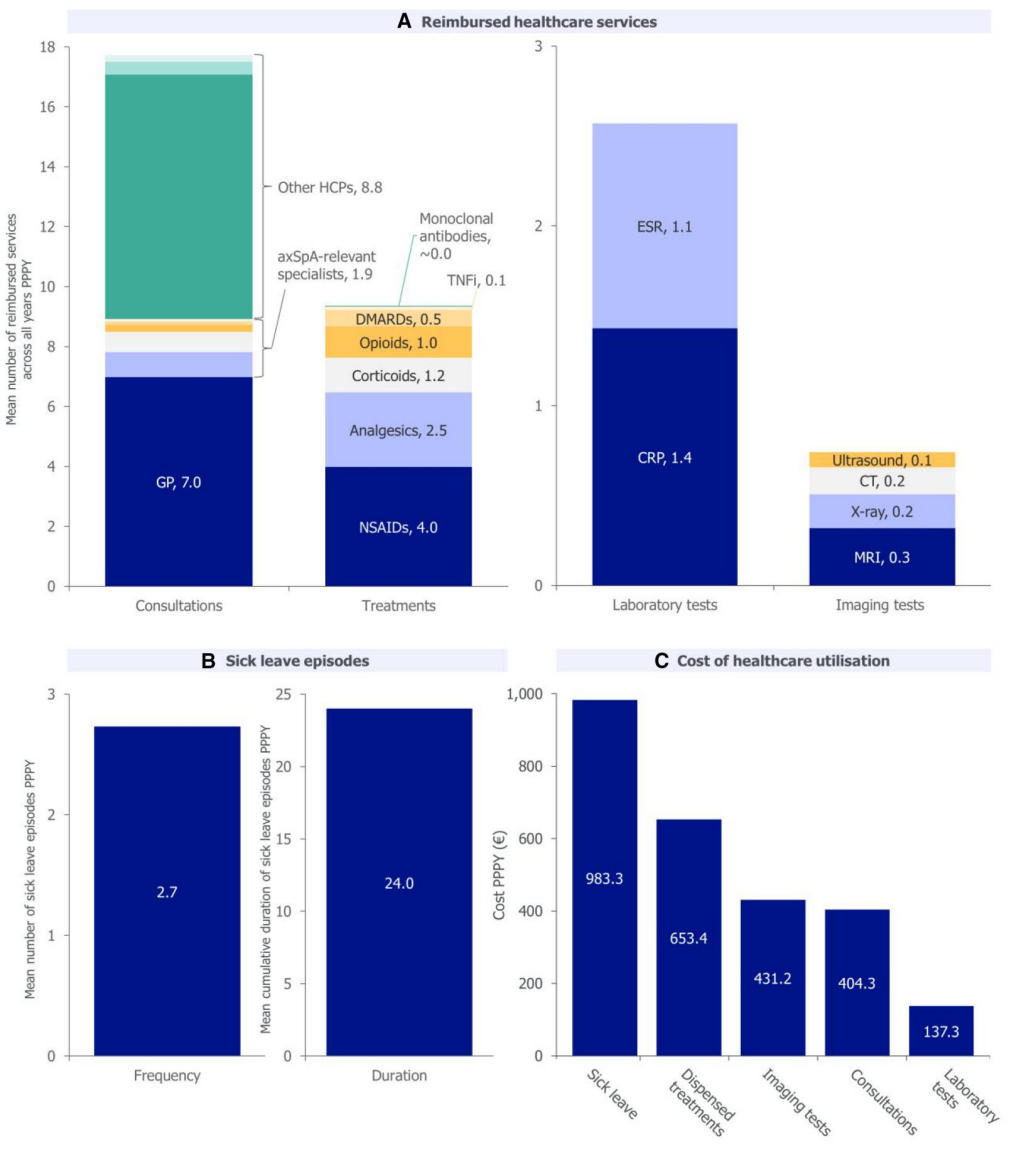
Consultations, treatments, laboratory tests, imaging tests and sick leave episodes prior to axSpA diagnosis. Reported for patients with back pain and any recorded reimbursement history prior to axSpA diagnosis (*N* = 1513). PPPY refers to the total cost of HCRU divided by the number of months of follow-up over the entire study period for all patients, multiplied by 12. Reimbursement data are only available on the THIN^®^ database from 2014 onwards. The sum of component values may not precisely match overall values due to rounding. Colours are used to differentiate categories, aiding in visual distinction rather than indicating equivalence. In panel A, axSpA-relevant specialists include rheumatologists (0.8), ophthalmologists (0.7) gastroenterologists (0.2), radiologists (0.1) and dermatologists (0.1); other HCPs include physiotherapists (8.2), mental health support workers (0.4) and osteopaths (0.2). In panel B, sick leave duration is cumulative across the year. In panel C, costs are reported PPPY for the time between earliest recorded back pain diagnosis and axSpA diagnosis. axSpA: axial spondyloarthritis; GP: general practitioner; HCP: healthcare professional; HCRU: healthcare resource utilization; PPPY: per patient per year; TNFi: tumour necrosis factor inhibitor

Mean number of HCP consultations (for any reason) PPPY between earliest back pain diagnosis and axSpA diagnosis was 7.0 for GPs, 1.9 for axSpA-relevant specialists and 8.8 for other HCPs (physiotherapists, mental health support workers and osteopaths) (Fig. 5A). Mean number of laboratory tests PPPY was 2.6, and mean number of imaging tests was 0.7 PPPY; MRI was the most common (Fig. 5A). A mean of 9.4 treatments were dispensed PPPY; NSAIDs and analgesics were the most common (Fig. 5A). Mean number of sick leave episodes was 2.7 PPPY, with a mean cumulative length of 24.0 days PPPY (Fig. 5B).

All HCRU variables increased as the year of axSpA diagnosis approached (Supplementary Fig. S3).

#### Costs

Mean cost associated with HCRU between earliest back pain diagnosis and axSpA diagnosis was €2609.5 PPPY; sick leave was the largest source (€983.3 PPPY; Fig. 5C). Associated costs across all HCRU variables were greatest in the year prior to axSpA diagnosis (Supplementary Table S6).

Mean cost for patients with a complete reimbursement history was €2658.3 PPPY.

### Time to diagnosis from earliest recorded back pain or axSpA-related feature

Among the axSpA indicators subgroup (*N* = 4826), mean (S.D.) time to axSpA diagnosis, based on the time since either the earliest axSpA-related feature or earliest back pain diagnosis (i.e. whichever came first), was 6.6 (4.5) years (Supplementary Fig. S4). Enthesitis was the most common, non-back pain, axSpA-related feature recorded prior to axSpA diagnosis, accounting for 8.0% of total diagnoses (Supplementary Fig. S5A). Enthesitis was also most frequently the earliest of the non-back pain, axSpA-related features recorded (11.7% of earliest diagnoses; Supplementary Fig. S5B).

Among patients with both back pain and axSpA-related feature diagnoses prior to axSpA diagnosis (*N* = 1430), time to diagnosis was 8.5 (4.4) years. 60.3% of patients experienced back pain first, 37.6% experienced axSpA-related features first and 2.1% had both recorded on the same date.

### Sensitivity analyses

#### Time to diagnosis among patients with chronic back pain

Between 1 January 2010 and 31 August 2023, 3012 patients experienced ‘chronic back pain’ (≥2 back pain codes recorded ≥90 days apart). In these patients, the mean (S.D.) time to diagnosis was 7.3 (4.3) years. About 46.0% of patients experienced a time to diagnosis >7 years (Supplementary Fig. S6).

#### Time to diagnosis among patients with no minimum or ≥7 years of continuous data

Mean time to diagnosis was also assessed in patient subgroups with a previous back pain diagnosis with no minimum requirement for continuous data prior to axSpA diagnosis (*N* = 5668) and a ≥7-year minimum requirement (*N* = 3064). Mean (S.D.) time to diagnosis in these groups was 5.1 (4.5) and 7.8 (4.5) years, respectively.

## Discussion

In this real-world study from a large French cohort, patients waited over 6 years for an axSpA diagnosis, highlighting that, despite efforts to improve early identification of axSpA [22], time to diagnosis remains a key challenge. The escalating burden of axSpA symptoms and comorbidities from earliest back pain diagnosis to axSpA diagnosis suggests that early detection may be beneficial.

This study identified that lower back pain and enthesitis were, respectively, the most common type of back pain and axSpA-related feature recorded before axSpA diagnosis. Interestingly, while ∼60% of patients initially experienced back pain, over 30% presented with other axSpA-related features first. This indicates that, although back pain is considered a typical symptom of axSpA [23], focusing on this alone may be insufficient for early diagnosis in some patients.

Analyses of patient characteristics indicated that the study cohort was generally comparable to previously-described axSpA cohorts in terms of sex distribution and high comorbidity burden [24–27]. However, the mean age at earliest back pain diagnosis (40 years) and at axSpA diagnosis (47 years) reported here was higher than previous reports [10, 12, 23, 24, 28]. Time to diagnosis (6.3 years) was also slightly lower than in previous studies (∼7 years) conducted over a similar time period (∼2008 to ∼2019) [12, 14]. This difference may reflect that, in this study, time to diagnosis was calculated using the time when earliest back pain and axSpA diagnoses were recorded by GPs, while previous studies have largely estimated time to diagnosis via patients self-reporting the time of first symptom onset. As it has been established that patients with axSpA often delay seeking consultations for their symptoms [18, 29], using GP-reported data in this study may have led to time to diagnosis being underestimated. However, patient-reported studies are prone to recall bias and therefore may lead to over- or underestimation of diagnostic delay [30].

This study found that the patient journey to axSpA diagnosis in France was associated with high HCRU. For example, the mean of 7.0 GP consultations PPPY was considerably higher than previously reported for the general French population between 2018 and 2019 (∼3) [31]. Patients also visited multiple specialists, consistent with previous studies [9, 12]. Furthermore, in this study, sick leave was a significant source of HCRU. Patients had a mean of 2.7 individual episodes of sick leave in the year prior to axSpA diagnosis, cumulating to 24.0 days PPPY. This duration is notably higher than the general French population, which was reported to be 8.2–8.9 days per year from 2010 to 2019 [32]. These findings are consistent with previous studies, where axSpA has been shown to significantly impact work productivity [33–35], particularly in patients experiencing longer diagnostic delay [9]. For example, in a German multicentre study of patients with axSpA, over 30% of employed patients reported ≥1 month of sick leave within the past year [34]. Treatment for axSpA has also been shown to improve work outcomes, including absences [34, 36].

The study findings are potentially generalizable to general practice in France in that the THIN database is representative of the French national population in terms of age, sex and region. However, when assessing the generalizability of the findings to the French axSpA population, the use of primary care data may have resulted in lower representation of patients with severe axSpA who are managed exclusively in secondary care. It is important to note that the findings discussed may not be generalizable beyond the French setting, as differences in national healthcare systems, such as referral pathways, access to specialists and reimbursement policies, can influence these outcomes. For example, HLA-B27 testing is not reimbursed in France, but is considered a valuable test to confirm diagnosis [37]. In other countries, HLA-B27 testing plays a crucial role in the timing of axSpA diagnosis; its absence in the reimbursement data in this case could influence both diagnosis and referral to a specialist.

The results of this study should be interpreted in the context of its limitations. As previously discussed, any delays between symptom onset and patients visiting their GP may have led to underestimation of time to diagnosis. Also, axSpA diagnoses made by rheumatologists or other secondary care services were contingent upon GPs documenting these retrospectively, potentially leading to imprecision in the recorded diagnosis date. Additionally, GPs may have faced challenges in accurately assigning certain axSpA symptoms to diagnostic codes within the THIN database, meaning some may have not been captured. Potential diagnostic miscoding is a common limitation associated with secondary healthcare data that is not exclusive to the THIN database [38–40].

Focusing on patients with any reimbursement history may have introduced selection bias, underestimating HCRU. However, focusing on patients with only complete reimbursement histories may have led to overestimation, and the any reimbursement subgroup was found to be more representative of the overall back pain subgroup. Additionally, overall HCRU-related costs PPPY of the two subgroups were similar (any reimbursement history: €2609.5 vs complete reimbursement history: €2658.3), indicating that the effect of this bias was minimal. Selection bias was also potentially introduced by requiring patients to have ≥3 years continuous data available prior to axSpA diagnosis, as this may exclude those with less stable healthcare access or recent changes in healthcare providers. While this is unlikely to affect the onset of axSpA itself, it is plausible that participants excluded due to shorter follow-up periods might have experienced different healthcare pathways. This could lead to earlier diagnosis following disease onset, potentially introducing bias in terms of time to diagnosis rather than onset. Indeed, sensitivity analysis demonstrated that time to diagnosis was lower with no minimum requirement for continuous data and was higher with a ≥7-year minimum. Although expected, this highlights the impact of data availability on estimating time to diagnosis.

Furthermore, focusing on the earliest instance of back pain, rather than chronic back pain, may have underestimated time to diagnosis by including patients with a single back pain episode shortly before axSpA diagnosis. However, >75% of patients included in the main back pain subgroup experienced >1 back pain episode and sensitivity analysis showed that the time to diagnosis was only slightly higher in the ‘chronic back pain’ subgroup. Finally, the lack of a control group (patients with back pain but without axSpA) prevented definitive attribution of the increase in axSpA symptoms, comorbidities and HCRU to delayed diagnosis, rather than another factor (i.e. ageing).

Strengths of this study included the large patient population, representative of the French patient landscape, derived from a high-quality, anonymized dataset that has been validated for the study of multiple diseases, including psoriasis and PsA [41, 42]. Additionally, by using electronic medical records, this study was not reliant on patient recall. As previously mentioned, recall bias is a common limitation in survey-based data, which many previous studies reporting time to diagnosis of axSpA have utilized [12, 15, 16].

## Conclusion

This study highlights the significant and sustained challenge of delayed axSpA diagnosis in in France, with a time to diagnosis of over 6 years. At the time of axSpA diagnosis, a higher proportion of patients experienced multiple axSpA symptoms and comorbidities than at earliest back pain diagnosis, and the diagnostic journey involved numerous consultations, tests and treatments. These findings may indicate that disease burden intensifies over time, underscoring the critical need for early detection and treatment initiation to mitigate this.

Ultimately, this research may help raise awareness amongst French primary care practitioners of the patient journey to axSpA diagnosis, and support development of primary care flagging strategies to identify patients with suspected axSpA who may benefit from a rheumatology referral. Such flagging strategies would help alleviate the burden of axSpA on patients and healthcare systems.

## Supplementary Material

keaf642_Supplementary_Data

## Data Availability

The data that support the findings of this study are available upon reasonable request to the corresponding author. The data are not publicly available due to privacy or ethical restrictions.
