# Supplementary material for: Time and patient journey to axial spondyloarthritis diagnosis: a retrospective study in French primary care
Source: Rheumatology (Oxford). 2026 Mar 15;65(3):keaf642. doi: 10.1093/rheumatology/keaf642 (PMC13017005; doi:10.1093/rheumatology/keaf642)
Supplement: keaf642_Supplementary_Data [file keaf642_supplementary_data.zip › Suppl Tables and Figures.docx]

SUPPLEMENTARY TABLES AND FIGURES

Table S1. List of axSpA diagnosis codes (BCB and ICD-10)

| **BCB code** | **BCB code diagnosis label in English** | **ICD-10 code** |
| --- | --- | --- |
| BCB.10005637 | Primary rheumatic pelvispondylitis | M45 |
| BCB.CIM10.M45 | Ankylosing spondylitis | M45 |
| BCB.AMM.0000005559 | Ankylosing spondylitis | M45 |
| BCB.10003505 | Rheumatic pelvispondylitis | M45 |
| BCB.47028 | Ankylosing spondylitis | M45 |
| BCB.10005540 | Ankylosing spondylitis | M45 |

BCB codes and the associated diagnosis labels are those utilised in the THIN^®^ database; corresponding ICD-10 codes are provided here. ICD-10 M45.Ax does not have any corresponding BCB codes, so this code is not utilised in the THIN^®^ database. axSpA: axial spondyloarthritis; BCB: Base Claude Bernard; ICD-10: International Classification of Diseases 10^th^ Revision; THIN: The Health Improvement Network.

Table S2. List of back pain diagnosis codes (BCB and ICD-10)

| **BCB code** | **BCB code diagnosis label in English** | **ICD-10 code** |
| --- | --- | --- |
| **Cervicalgia** | | |
| BCB.10003826 | Cervicalgia | M54.2 |
| BCB.10005702 | Cervicalgia | M54.2 |
| BCB.10005703 | Chronic cervicalgia | M54.2 |
| BCB.10005704 | Chronic cervicalgia due to posterior joint cause | M54.2 |
| BCB.10005705 | Cervicalgia due to musculoskeletal cause | M54.2 |
| BCB.10008255 | Cervical pain | M54.2 |
| BCB.10036079 | Cervicodorsalgia | M54.8 |
| BCB.47007 | Cervicalgia | M54.2 |
| BCB.69253 | Cervical pain | M54.2 |
| BCB.10036303 | Neck pain | M54.2 |
| BCB.10005826 | Cervicarthrosis with radiculopathy | M47.2 |
| BCB.CIM10.M47.2 | Other spondyloarthroses with radiculopathy | M47.2 |
| BCB.CIM10.M50.1 | Cervical disc disease with radiculopathy | M50.1 |
| BCB.14048 | Cervicobrachial neuralgia (CBN) | M53.1 |
| BCB.42013 | Cervicobrachial neuralgia (CBN) | M53.1 |
| BCB.10005706 | Cervicobrachial neuralgia (CBN) | M53.1 |
| BCB.10018293 | Cervicobrachial pain | M53.1 |
| BCB.10036282 | Right cervicobrachialgia | M53.1 |
| BCB.AMM.0000004600 | Cervicobrachial neuralgia | M53.1 |
| BCB.AMM.0000005244 | Cervicobrachial radiculalgia | M53.1 |
| BCB.CIM10.M53.0 | Cervico-cephalic syndrome | M53.0 |
| BCB.CIM10.M53.1 | Cervicobrachial syndrome | M53.1 |
| BCB.10002311 | Narrow cervical canal | M53.8 |
| **Dorsalgia/Rachialgia** | | |
| BCB.10003827 | Dorsalgia | M54.9 |
| BCB.10005711 | Musculoskeletal dorsal pain | M54.9 |
| BCB.10005713 | Visceral dorsal pain | M54.9 |
| BCB.10010409 | Post-traumatic dorsal pain | M54.9 |
| BCB.10011195 | Dorsal pain | M54.9 |
| BCB.10035488 | Acute dorsal pain | M54 |
| BCB.20261 | Dorsal pain | M54.9 |
| BCB.47011 | Dorsalgia | M54.9 |
| BCB.69045 | Dorsal pain | M54.9 |
| BCB.10005714 | Dorsalgia | M54.9 |
| BCB.10008254 | Spinal pain | M54.9 |
| BCB.80000725 | Dorsal pain | M54.9 |
| BCB.10003794 | Rachialgia | M54.9 |
| BCB.10005841 | Diffuse rachialgia | M54.9 |
| BCB.10008078 | Spinal blockage-stiffness | M53.8 |
| BCB.10008328 | Spine pain | M54.9 |
| BCB.10009452 | Spine pain | M54.9 |
| BCB.10010856 | Rachialgia | M54.9 |
| BCB.10036458 | Modic 1 disc disease | M51.9 |
| BCB.10005828 | Dorsarthrosis with radiculopathy | M47.2 |
| BCB.CIM10.M53.9 | Unspecified dorsopathy | M53.9 |
| BCB.CIM10.M53.8 | Other specified dorsopathies | M53.8 |
| BCB.CIM10.M51 | Involvement of other intervertebral discs | M51 |
| BCB.10013138 | Pinching of intervertebral discs | M51.8 |
| BCB.10010859 | Disc disease | M51.9 |
| BCB.10003916 | Vertebral instability | M53.2 |
| BCB.CIM10.M53.2 | Vertebral instability | M53.2 |
| BCB.CIM10.M51.8 | Other specified intervertebral disc disorders | M51.8 |
| BCB.CIM10.M51.9 | Unspecified intervertebral disc damage | M51.9 |
| **Lombalgia** | | |
| BCB.10003932 | Low back pain | M54.5 |
| BCB.10004276 | Chronic low back pain | M54.5 |
| BCB.10004372 | Acute low back pain | M54.5 |
| BCB.10005715 | Low back pain | M54.5 |
| BCB.10005716 | Chronic low back pain due to posterior joint cause | M54.5 |
| BCB.10005725 | Chronic low back pain due to musculoskeletal cause | M54.5 |
| BCB.10010445 | Nocturnal low back pain | M54.5 |
| BCB.10035489 | Dorso-lumbalgia | M54.8 |
| BCB.10036308 | Common vertebral low back pain | M54.5 |
| BCB.24133 | Chronic low back pain | M54.5 |
| BCB.47018 | Acute low back pain | M54.5 |
| BCB.47019 | Chronic low back pain | M54.5 |
| BCB.47097 | Low back pain | M54.5 |
| BCB.69259 | Low back pain | M54.5 |
| BCB.74797 | Low back pain | M54.5 |
| BCB.10004275 | Lumbago | M54.5 |
| BCB.10008782 | Low back pain | M54.5 |
| BCB.10010654 | Initial low back pain | M54.5 |
| BCB.10010656 | Unilateral low back pain | M54.5 |
| BCB.10034863 | Acute lumbago | M54.5 |
| BCB.17070 | Low back pain | M54.5 |
| BCB.20070 | Low back pain | M54.5 |
| BCB.24132 | Lumbago | M54.5 |
| BCB.47099 | Lumbago | M54.5 |
| BCB.70070 | Low back pain | M54.5 |
| BCB.80000482 | Dorsalgia | M54.5 |
| BCB.10004437 | Lumbar disc disease | M51.3 |
| BCB.10005830 | Lumbar osteoarthritis with radiculopathy | M47.2 |
| BCB.10035593 | Lumbar stiffness | M53.8 |
| BCB.10002310 | Narrow lumbar canal | M53.8 |
| BCB.CIM10.M51.1 | Lumbar and other intervertebral discs with radiculopathy | M51.1 |
| BCB.10005440 | Acquired narrowed lumbar canal of posterior articular arthrosic origin | M53.8 |
| BCB.10036036 | L4-L5 disc collapse | M51.3 |
| BCB.CIM10.M51.0 | Involvement of lumbar and other intervertebral discs with myelopathy | M51.0 |
| BCB.10010444 | Lombofessalgie' |  |
| BCB.24136 | Lumbosciatica | M54.4 |
| BCB.10004380 | Lumbosciatica | M54.4 |
| BCB.10005718 | Lumbosciatica due to disc herniation | M51.1 |
| BCB.10005719 | Recurrent lumbago after disc surgery | M51.1 |
| BCB.10005722 | Lumbosciatica due to lumbar canal stenosis | M54.4 |
| BCB.10005723 | Lumbosciatica due to posterior articular cause (cyst) | M54.4 |
| BCB.AMM.0000004224 | Lombosciatica | M54.4 |
| BCB.10039 | Traumatic lesion of the lumbosacral plexus |  |
| BCB.60039 | Traumatic lesion of the lumbosacral plexus |  |
| BCB.10002063 | Postradial lumbosacral plexitis | G54.1 |
| BCB.10002658 | Lumbosacral plexus disorders | G54.1 |
| BCB.10002661 | Lumbosacral root disorders | G54.4 |
| BCB.CIM10.G54.1 | Lumbosacral plexus disorders | G54.1 |
| BCB.CIM10.G54.4 | Lumbosacral root disorders, not elsewhere classified | G54.4 |
| **Coccyx/sacralgia** | | |
| BCB.10013139 | Pinching of the L5-S1 intervertebral disc | M51.3 |
| BCB.10005848 | Sacralization of fifth lumbar vertebra (incomplete) | Q76.4 |
| BCB.10004295 | Hemisacralization | Q76.4 |
| BCB.10004296 | Sacralization | Q76.4 |
| BCB.10005835 | Sacralgia (Sacrodynia) | M53.3 |
| BCB.10005848 | Sacralization of the fifth lumbar vertebra (incomplete) | Q76.4 |
| BCB.24380 | Sacroiliitis | M46.1 |
| BCB.10003531 | Sacroiliitis | M46.1 |
| BCB.10006435 | Crohn's disease with sacroiliitis | K50.9 |
| BCB.10035217 | Sacroiliac pain | M25.5 |
| BCB.CIM10.M46.1 | Sacroiliitis, not elsewhere classified | M46.1 |
| BCB.10035749 | Iliac wing pain | M53.3 |
| BCB.10003964 | Sacro-coccygeal fossa | M53.3 |
| BCB.10016761 | Sacro-coccygeal pain | M53.3 |
| BCB.CIM10.M53.3 | Sacro-coccygeal involvement, not elsewhere classified | M53.3 |
| BCB.24502 | Coccygodynia (Coccydynia) | M53.3 |
| BCB.47078 | Coccygodynia | M53.3 |
| BCB.10003795 | Coccygodynia | M53.3 |
| BCB.10008322 | Coccyx pain (Coccygodynia) | M53.3 |
| BCB.10034652 | Coccydynia | M53.3 |
| BCB.10008078 | Spinal blockage – stiffness | M53.8 |
| BCB.AMM.0000024896 | Radiculopathy | M54.1 |
| BCB.CIM10.M54.1 | Radiculopathy | M54.1 |
| BCB.10035947 | Retrolisthesis | M53.2 |
| **Buttock pain** | | |
| BCB.20604 | Buttock pain | M54.8 |
| BCB.10003981 | Buttock pain | M54.8 |
| **Sciatica** | | |
| BCB.10037 | Sciatica L4 |  |
| BCB.14513 | Sciatica L5 | M54.3 |
| BCB.14514 | Sciatica S1 | M54.3 |
| BCB.21176 | Sciatica | M54.3 |
| BCB.24500 | Sciatica L5 | M54.3 |
| BCB.24501 | Sciatica S1 | M54.3 |
| BCB.47026 | Sciatica | M54.3 |
| BCB.47051 | Sciatica L5 | M54.3 |
| BCB.47052 | Sciatica S1 | M54.3 |
| BCB.60087 | Sciatica L5 |  |
| BCB.73982 | Sciatica |  |
| BCB.74836 | Sciatica | Z00.0 |
| BCB.81089 | Sciatica |  |
| BCB.10001878 | Sciatica L5 | M54.3 |
| BCB.10001879 | Sciatica S1 | M54.3 |
| BCB.10002407 | Hip and thigh sciatic nerve injury | S74.0 |
| BCB.10002677 | Other sciatic nerve injury | G57.0 |
| BCB.10003920 | Truncated sciatica | M54.3 |
| BCB.10004278 | Sciatica | M54.3 |
| BCB.10004279 | Paralytic sciatica | M54.3 |
| BCB.10005726 | Truncal sciatica | G54.0 |
| BCB.10005843 | Sciatica, unspecified |  |
| BCB.10035621 | Bilateral sciatica | M54.3 |
| BCB.AMM.0000005430 | Sciatica | M54.3 |
| BCB.AMM.0000018657 | Sciatic algodystrophy |  |
| BCB.CIM10.G57.0 | Sciatic nerve injury | G57.0 |
| BCB.CIM10.M54.3 | Sciatica | M54.3 |
| BCB.CIM10.M54.4 | Lumbago with sciatica | M54.4 |
| BCB.CIM10.S74.0 | Traumatic sciatic nerve injury at hip and thigh | S74.0 |

BCB codes and the associated diagnosis labels are those utilised in the THIN^®^ database; corresponding ICD-10 codes are provided here. axSpA: axial spondyloarthritis; BCB: Base Claude Bernard; ICD-10: International Classification of Diseases 10^th^ Revision; THIN: The Health Improvement Network.

Table S3. Demographics and characteristics among patients with no recorded back pain prior to axSpA diagnosis

|  | **Patients with no back pain prior to axSpA diagnosis**  **(N=2,911)** |  |
| --- | --- | --- |
| **Age**, years, mean (SD) | | 48 (15) |
| **Sex**, n (%) | |  |
| Male | | 1,384 (47.5) |
| Female | | 1,527 (52.5) |
| **Length of continuous medical history**,^a^ years, mean (SD) | | 8.7 (4.6) |
| **CCI**, n (%) | |  |
| 0 | | 1,277 (43.9) |
| 1–2 | | 1,131 (38.9) |
| 3–4 | | 365 (12.5) |
| >4 | | 138 (4.7) |
| **>1 diagnosis of a comorbidity or symptom of axSpA**, n (%) | | 1,151 (39.5) |
| **Most common comorbidities or symptoms of axSpA**, n (%) | |  |
| Hypertension | | 576 (19.8) |
| Depression | | 450 (15.5) |
| Fatigue | | 526 (18.1) |

[a] Refers to the length of continuous medical history available in the THIN^®^ database, following back from the date of axSpA diagnosis (i.e. index date). axSpA: axial spondyloarthritis; CCI: Charlson Comorbidity Index; SD: standard deviation.

**Table S4.** List of enthesitis codes investigated as symptoms of axSpA (ICD-10)

| **ICD-10 code** |
| --- |
| M77.00, M77.01, M77.02, M77.10, M77.11, M77.12, M76.00, M76.01, M76.02, M76.50, M76.51, M76.52, M76.60, M76.61, M76.62, M76.891, M76.892, M76.899, M76.9, M77.50, M77.51, M77.52, M77.8, M77.9 |

axSpA: axial spondyloarthritis; ICD-10: International Classification of Diseases 10th Revision.

Table S5. Demographics among patients with a complete reimbursement history and patients with any reimbursement history

|  | **Patients with a complete reimbursement history (N=402)** | | **Patients with any reimbursement history (N=1,513)** | |
| --- | --- | --- | --- | --- |
|  | **At back pain diagnosis** | **At axSpA diagnosis** | **At back pain diagnosis** | **At axSpA diagnosis** |
| **Age**, years, mean (SD) | 41.3 (13.7) | 45.9 (14.5) | 39.5 (13.8) | 46.7 (14.3) |
| **Sex**, n (%) |  |  |  |  |
| Male | 154 (38%) | 154 (38%) | 597 (39%) | 597 (39%) |
| Female | 248 (62%) | 248 (62%) | 916 (61%) | 916 (61%) |

Patients with ≥5 years of reimbursement history prior to axSpA diagnosis were considered to have a ‘complete’ reimbursement history. Patients with ≥1 request from the GP of reimbursement history, including patients with a ‘complete’ reimbursement history, were considered to have ‘any’ reimbursement history. axSpA: axial spondyloarthritis; SD: standard deviation.

Table S6. Cost of healthcare resource utilisation prior to axSpA diagnosis

|  | **Associated cost**, PPPY,^a^ € (% of overall cost) | | | | | |
| --- | --- | --- | --- | --- | --- | --- |
| **Year before axSpA diagnosis** | **Overall** | **Sick leave** | **Treatments** | **Imaging tests** | **Consultations** | **Laboratory tests** |
| **Mean across all years (N=1,513)** | **2,609.5 (100)** | **983.3 (37.7)** | **653.4 (25.0)** | **431.2 (16.5)** | **404.3 (15.5)** | **137.3 (5.3)** |
| **0–1** (N=1,513) | 3,229.7 (100.0) | 1,317.7 (40.8) | 853.8 (26.4) | 456.2 (14.1) | 431.5 (13.4) | 170.6 (5.3) |
| **1–2** (N=894) | 2,371.5 (100.0) | 878.7 (37.1) | 590.8 (24.9) | 385.4 (16.3) | 386.4 (16.3) | 130.2 (5.5) |
| **2–3** (N=575) | 2,016.7 (100.0) | 703.2 (34.9) | 471.0 (23.4) | 368.7 (18.3) | 367.2 (18.2) | 106.7 (5.3) |
| **3–4** (N=359) | 1,884.2 (100.0) | 724.9 (38.5) | 422.6 (22.4) | 298.5 (15.8) | 348.6 (18.5) | 89.7 (4.8) |
| **4–5** (N=224) | 1,661.7 (100.0) | 465.1 (28.0) | 465.9 (28.0) | 300.9 (18.1) | 340.5 (20.5) | 89.2 (5.4) |
| **5–6** (N=117) | 1,445.0 (100.0) | 427.9 (29.6) | 316.0 (21.9) | 288.6 (20.0) | 337.4 (23.4) | 75.1 (5.2) |

Reported for patients with back pain and any recorded reimbursement history prior to axSpA diagnosis (N=1,513). [a] The mean cost divided by the number of months of follow-up over the entire study period for all patients and multiplied by 12. Reimbursement data are only available on the THIN^®^ database from 2014 onwards. axSpA: axial spondyloarthritis; PPPY: per patient per year.

**Figure S1.** Patient flow diagram


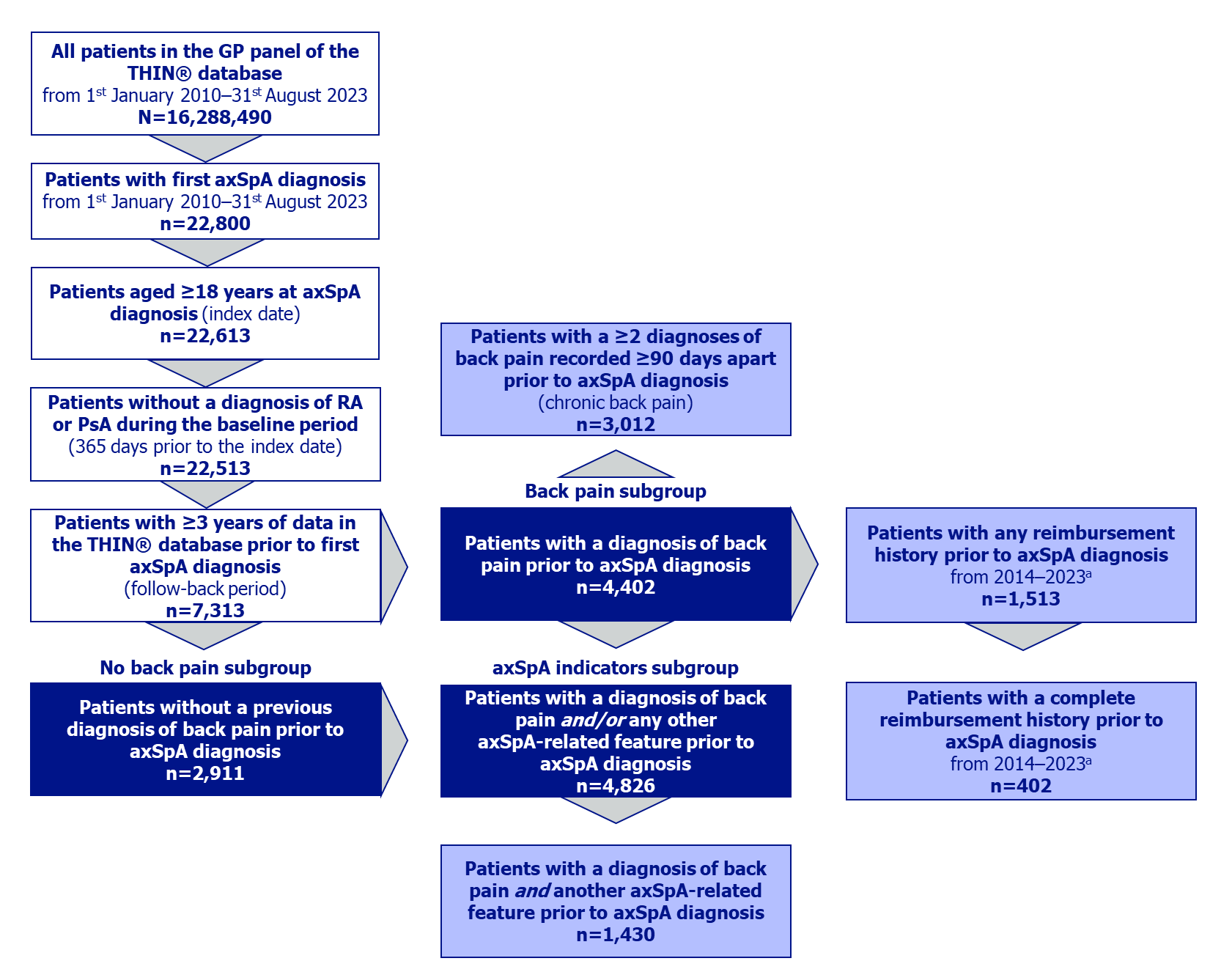


[a] Reimbursement data are only available from 2014 onwards. axSpA: axial spondyloarthritis; GP: general practitioner; PsA: psoriatic arthritis; RA: rheumatoid arthritis; THIN: The Health Improvement Network.

Figure S2. Non-back pain-related symptoms of axSpA and comorbidities at earliest back pain diagnosis and at axSpA diagnosis, for patients with a complete or any reimbursement history


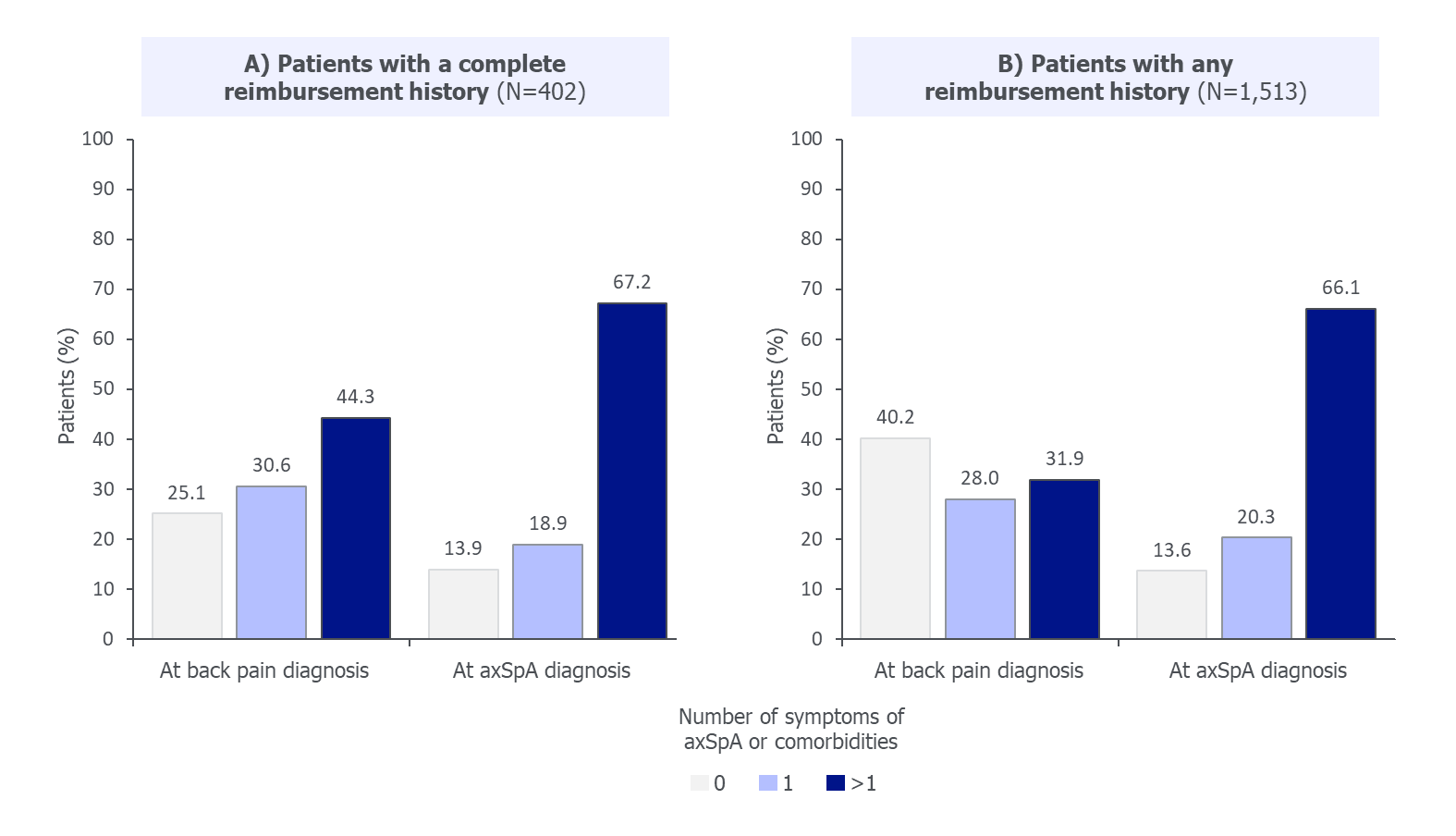


Symptoms of axSpA reported here do not include those related to back pain. Patients with ≥5 years of reimbursement history prior to axSpA diagnosis were considered to have a ‘complete’ reimbursement history. Patients with ≥1 request from the GP of reimbursement history, including patients with a ‘complete’ reimbursement history, were considered to have ‘any’ reimbursement history. Reimbursement data are only available on the THIN^®^ database from 2014 onwards. axSpA: axial spondyloarthritis.

**Figure S3.** Consultations, laboratory tests, imaging tests, treatments and sick leave episodes prior to axSpA diagnosis


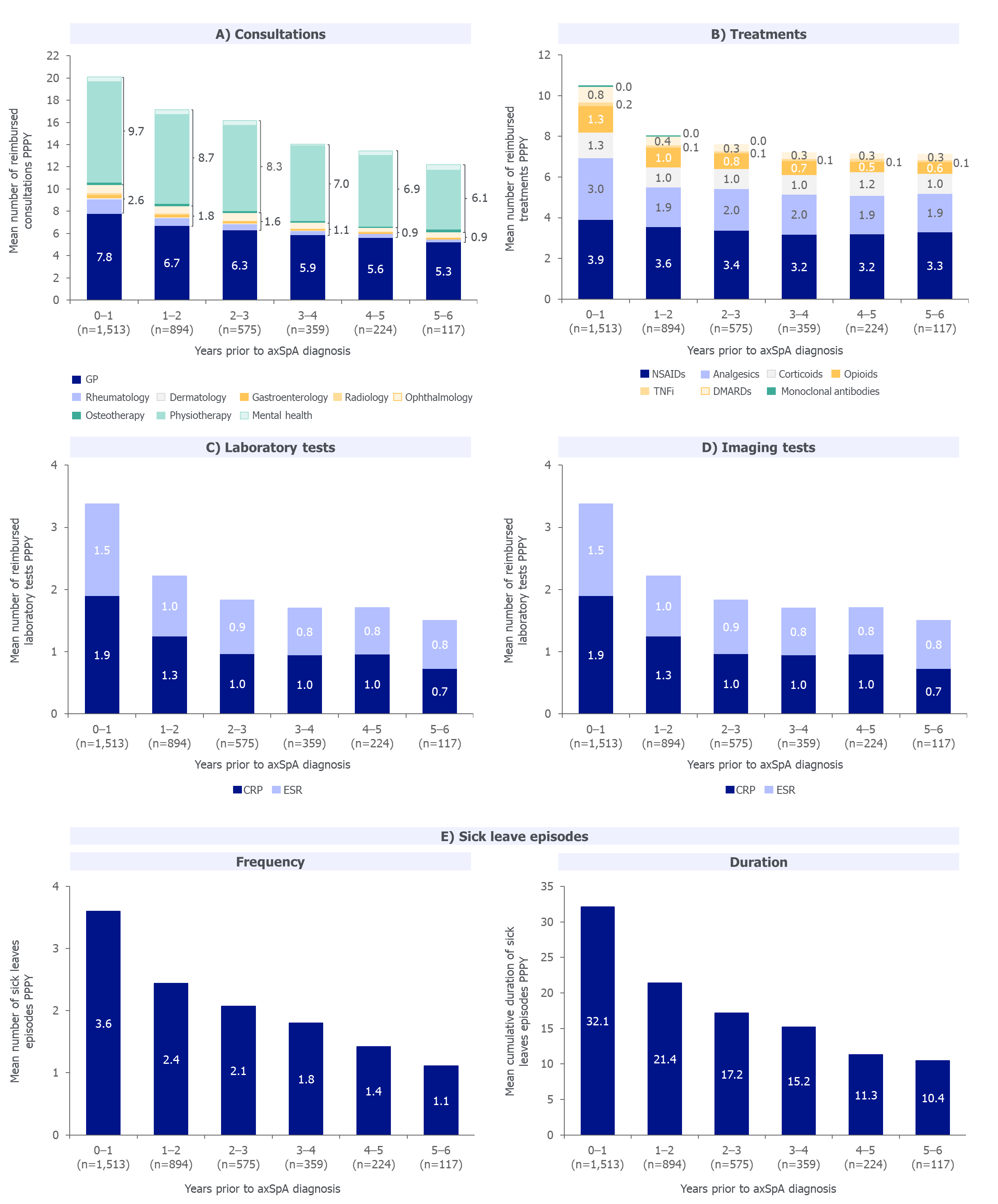


Reported for patients with back pain and any recorded reimbursement history prior to axSpA diagnosis (N=1,513). PPPY refers to the total cost of HCRU divided by the number of months of follow-up over the entire study period for all patients, multiplied by 12. Sick leave duration is cumulative across the year. Reimbursement data are only available on the THIN® database from 2014 onwards. The sum of component values may not precisely match overall values due to rounding. axSpA: axial spondyloarthritis; CRP: C-reactive protein; CT: computed tomography; DMARD: disease-modifying anti-rheumatic drug; ESR: erythrocyte sedimentation rate; GP: general practitioner; HCRU: healthcare resource utilisation; MRI: magnetic resonance imaging; NASAID: non-steroidal anti-inflammatory drugs; PPPY: per patient per year; TNFi: tumour necrosis factor inhibitor.

Figure S4. Time to diagnosis among the axSpA indicators subgroup


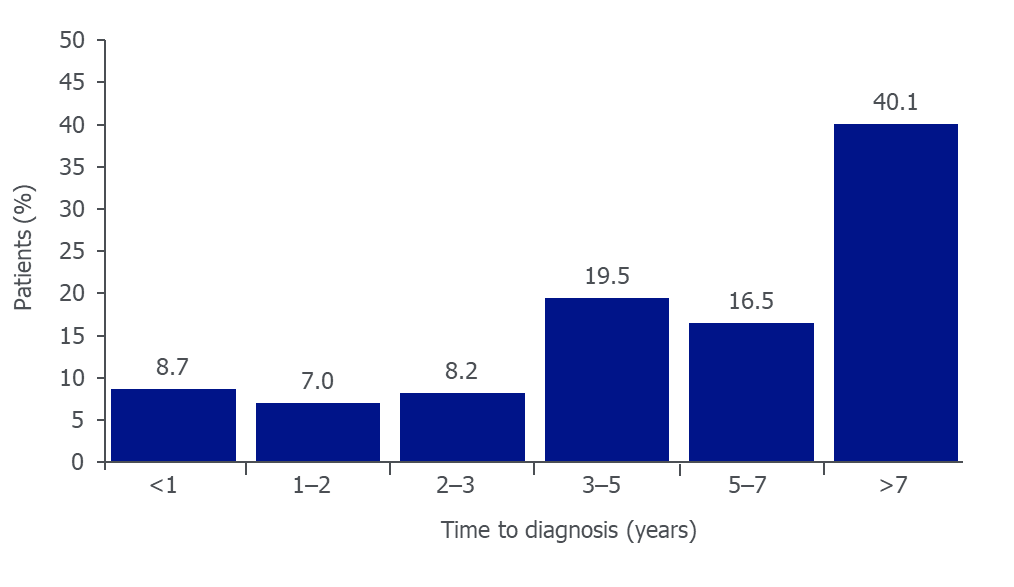


Reported for the axSpA indicators subgroup (N=4,826). Time to diagnosis was calculated based on the length of time to axSpA diagnosis from the earliest documented axSpA-related feature diagnosis or back pain diagnosis. axSpA-related features were defined as those described in the Assessment of Spondyloarthritis International Society (ASAS) classification criteria and included peripheral arthritis, enthesitis, dactylitis uveitis, psoriasis, and inflammatory bowel disease. axSpA: axial spondyloarthritis.

Figure S5. Distribution of location and type of all and earliest back pain or axSpA-related feature diagnoses prior to axSpA diagnosis


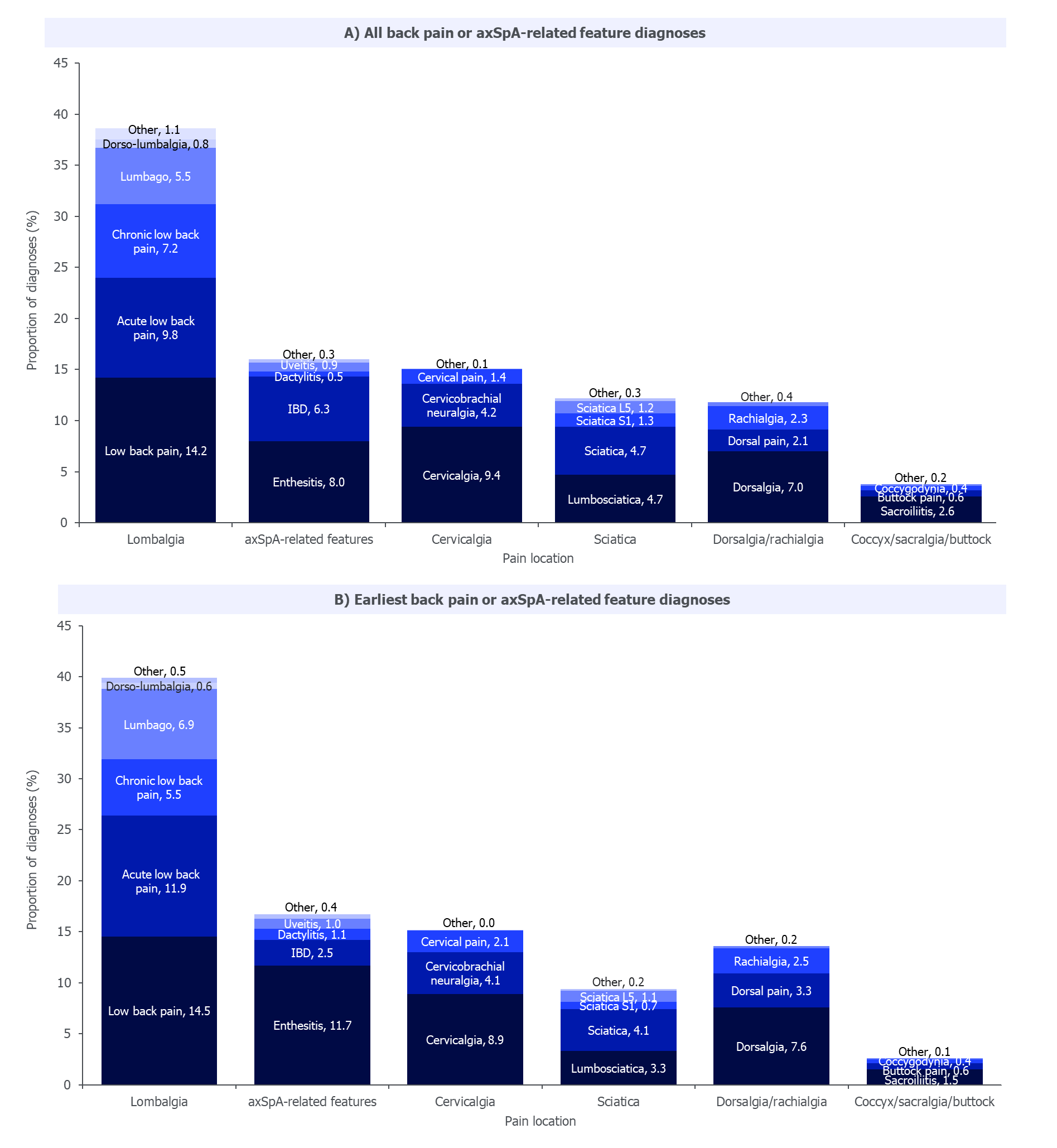


Reported for the axSpA indicators subgroup (N=4,826). Data are reported as (A) a proportion of the total number of back pain or axSpA-related feature diagnoses (N=37,632) and (B) a proportion of the total number of earliest back pain or axSpA-related feature diagnoses (N=5,343). A single patient could have two ‘earliest’ diagnoses on the same date. axSpA: axial spondyloarthritis; IBD: inflammatory bowel disease.

**Figure S6.** Time to diagnosis among patients experiencing chronic back pain


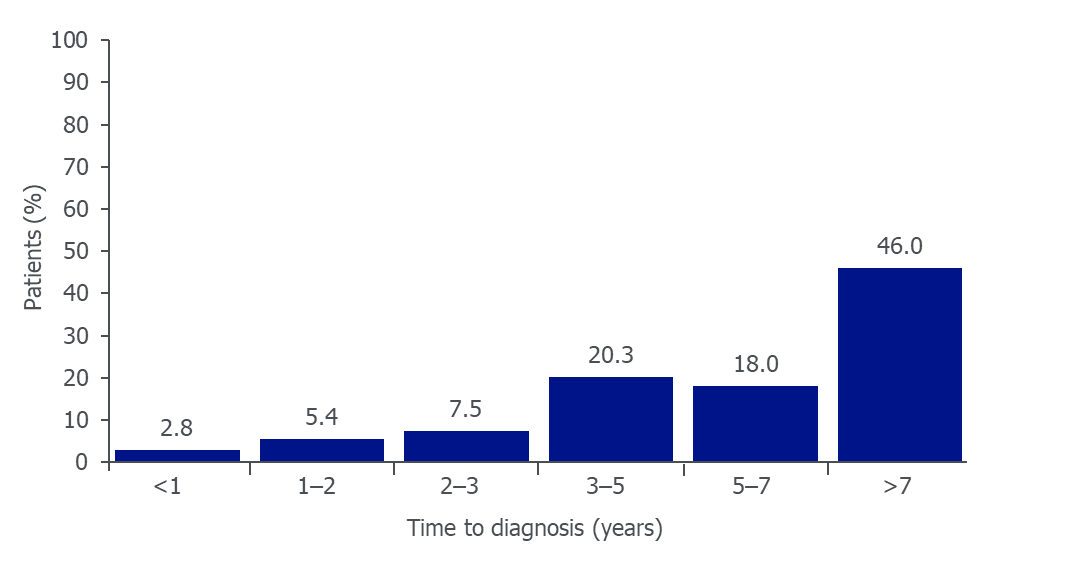


Reported for patients with chronic back pain (N=3,012). Time to diagnosis was calculated based on the length of time to axSpA diagnosis from the earliest documented back pain diagnosis. Chronic back pain was defined as patients with two back pain codes recorded with a minimum of 90 days apart. axSpA: axial spondyloarthritis.
