# Supplementary material for: Time and patient journey to axial spondyloarthritis diagnosis: a retrospective study in French primary care
Source: Rheumatology (Oxford). 2026 Mar 15;65(3):keaf642. doi: 10.1093/rheumatology/keaf642 (PMC13017005; doi:10.1093/rheumatology/keaf642)
Supplement: keaf642_Supplementary_Data [file keaf642_supplementary_data.zip › Plain Language Summary.docx]

PLAIN LANGUAGE SUMMARY

What is axial spondyloarthritis?

Axial spondyloarthritis (axSpA) is a disease that inflames the joints of the spine and hips of around 5 in every 1,000 people. People living with axSpA often experience back pain, stiffness, tiredness and lower quality of life. Diagnosing and treating axSpA early is essential to slow down or stop the disease from getting worse. However, people with axSpA often have to wait several years for a correct diagnosis.

What did we do?

In this study, our goal was to find out how long people wait for an axSpA diagnosis in France. We also aimed to learn more about how axSpA affects patients before they are diagnosed.

To do this, we looked at French general practitioner (GP) records from 2000 to 2023. Focusing on the gap between a patient’s first GP visit for back pain and when they were later diagnosed with axSpA, we asked:

1. How long was the gap?
2. How often did they visit their GP for back pain, and what type of back pain did they have?
3. What other axSpA symptoms or health problems did they have?
4. How much were healthcare services used?

What did we find?

After looking at the GP records, we found:

1. Patients in France wait a long time for an axSpA diagnosis, with a gap of over 6 years on average from when they first visited their GP.
2. Almost 1 in 5 patients reported back pain more than 10 times before their axSpA diagnosis, most often in the lower back.
3. More patients had multiple axSpA symptoms or other health problems when they were diagnosed with axSpA compared to when they first had back pain.
4. Patients used healthcare services often before their axSpA diagnosis. For example, patients visited their GP 7 times each year on average and had lots of tests and different types of treatment.

Why is this important?

Our findings show that delayed diagnosis is a key challenge for people with axSpA in France. Our study may help to raise awareness of the difficulties people face on their journey to an axSpA diagnosis. It may also help with creating tools that GPs can use to identify people who have axSpA earlier. This could allow them to access effective care sooner and ultimately have better control of their disease.
